# (*E*)-3-(3,5-Dimethoxy­phen­yl)acrylo­hydrazide

**DOI:** 10.1107/S1600536808028985

**Published:** 2008-09-13

**Authors:** Shahzad Ahmed, Ghulam Qadeer, Nasim Hasan Rama, Ales Ruzicka

**Affiliations:** a Department of Chemistry, Quaid-i-Azam Univeristy, Islamabad 45320, Pakistan; b Department of General and Inorganic Chemistry, Faculty of Chemical Technology, University of Pardubice, Nam. Cs. Legii’ 565, 53210 Pardubice, Czech Republic

## Abstract

In the title compound, C_11_H_14_N_2_O_3_, the planar hydrazide group is oriented with respect to the benzene ring at a dihedral angle of 48.00 (3)°. In the crystal structure, inter­molecular N—H⋯O hydrogen bonds link the mol­ecules.

## Related literature

For related literature, see: Zheng *et al.* (2003 ▶); Al-Talib *et al.* (1990 ▶); Yousif *et al.* (1986 ▶); Ahmad *et al.* (2001 ▶); Al-Soud *et al.* (2004 ▶); El-Emam *et al.* (2004 ▶); Furniss *et al.* (1978 ▶). For bond-length data, see: Allen *et al.* (1987 ▶).
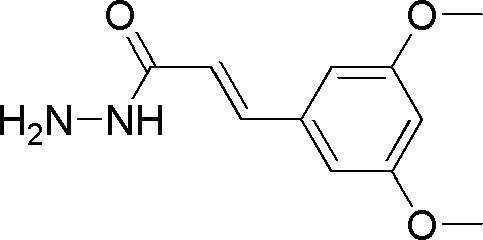


## Experimental

### 

#### Crystal data

C_11_H_14_N_2_O_3_
*M* *_r_* = 222.24
Monoclinic, 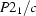

*a* = 4.8910 (19) Å
*b* = 30.358 (11) Å
*c* = 8.3440 (14) Å
β = 113.02 (3)°
*V* = 1140.4 (7) Å^3^
*Z* = 4
Mo *K*α radiation
μ = 0.10 mm^−1^
*T* = 150 (1) K
0.90 × 0.17 × 0.12 mm

#### Data collection

Bruker–Nonius KappaCCD area-detector diffractometer
Absorption correction: gaussian (Coppens, 1970 ▶) *T*_min_ = 0.961, *T*_max_ = 0.993
7864 measured reflections
2522 independent reflections
1547 reflections with *I* > 2σ(*I*)
*R*_int_ = 0.139

#### Refinement

*R*[*F* ^2^ > 2σ(*F* ^2^)] = 0.111
*wR*(*F* ^2^) = 0.274
*S* = 1.13
2522 reflections
145 parameters
H-atom parameters constrained
Δρ_max_ = 0.33 e Å^−3^
Δρ_min_ = −0.41 e Å^−3^

### 

Data collection: *COLLECT* (Hooft, 1998 ▶) and *DENZO* (Otwinowski & Minor, 1997 ▶); cell refinement: *DIRAX*/*LSQ* (Duisenberg, 1992 ▶); data reduction: *EvalCCD* (Duisenberg, 1992 ▶); program(s) used to solve structure: *SIR92* (Altomare *et al.*, 1994 ▶); program(s) used to refine structure: *SHELXL97* (Sheldrick, 2008 ▶); molecular graphics: *PLATON* (Spek, 2003 ▶); software used to prepare material for publication: *SHELXL97*.

## Supplementary Material

Crystal structure: contains datablocks I. DOI: 10.1107/S1600536808028985/hk2530sup1.cif

Structure factors: contains datablocks I. DOI: 10.1107/S1600536808028985/hk2530Isup2.hkl

Additional supplementary materials:  crystallographic information; 3D view; checkCIF report

## Figures and Tables

**Table 1:** Hydrogen-bond geometry (Å, °)

*D*—H⋯*A*	*D*—H	H⋯*A*	*D*⋯*A*	*D*—H⋯*A*
N1—H1⋯O1^i^	0.86	2.02	2.870 (3)	168

Symmetry code: (i) 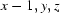.

**Fig. 1.**
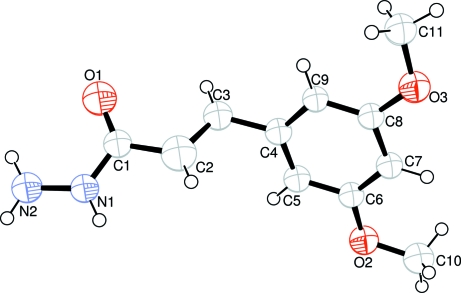
The molecular structure of the title molecule with the atom-numbering scheme.

**Fig. 2.**
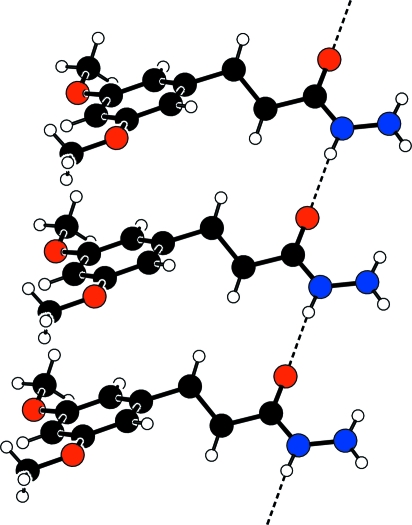
A packing diagram of the title compound. Hydrogen bonds are shown as dashed lines.

**Fig. 3.**
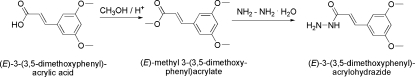
Reaction scheme.

## supplementary crystallographic information

### Comment

Aromatic hydrazides are important intermediates in heterocyclic chemistry and have been used for the synthesis of various biologically active five-membered heterocycles such as 2,5-disubstituted-1,3,4-oxadiazoles (Zheng *et al.*, 2003; Al-Talib *et al.*, 1990) and 5-substituted-2-mercapto-1,3,4-oxadiazoles (Yousif *et al.*,  1986; Ahmad *et al.*,  2001; Al-Soud *et al.*, 2004; El-Emam *et al.*, 2004). In view of the versatility of these compounds, we have synthesized the title compound, and report herein its crystal structure.

In the molecule of the title compound, (Fig. 1), the bond lengths (Allen *et al.*,  1987) and angles are generally within normal ranges. The benzene ring (C4-C9) is oriented with respect to the planar hydrazide group (O1/N1/N2/C1) at a dihedral angle of 48.00 (3)°.

In the crystal structure, intermolecular N-H···O hydrogen bonds (Table 1) link the molecules (Fig. 2), in which they may be effective in the stabilization of the structure.

### Experimental

The title compound was synthesized by the reaction of methyl ester of (E)-3 -(3,5-dimethoxyphenyl)acrylic acid with hdyrazine hydrate according to the literature method (Furniss *et al.*,  1978). For the preparation of the title compound, a mixture of (E)-methyl 3-(3,5-dimethoxyphenyl)acrylate (2.22 g, 10 mmol) and hydrazine hydrate (15 ml, 80%) in absolute ethanol (50 ml) was refluxed for 5 h at 413-423 K. The excess solvent was removed by distillation. The solid residue was filtered off, washed with water and recrystallized from ethanol (30%) to give the title compound (yield; 1.55 g, 70%, m.p. 401-402 K). Colorless single crystals were obtained by slow evaporation of an ethanol solution at room temperature.

### Refinement

H atoms were positioned geometrically, with N-H = 0.86 Å (for NH and NH_2_) and C-H = 0.93 and 0.96 Å for aromatic and methyl H, respectively, and constrained to ride on their parent atoms with U_iso_(H) = 1.2U_eq_(C,N).

### Crystal data

**Table:**

C_11_H_14_N_2_O_3_	*F*(000) = 472
*M**_r_* = 222.24	*D*_x_ = 1.294 Mg m^−^^3^
Monoclinic, *P*2_1_/*c*	Melting point: 401(1) K
Hall symbol: -P 2ybc	Mo *K*α radiation, λ = 0.71073 Å
*a* = 4.8910 (19) Å	Cell parameters from 7914 reflections
*b* = 30.358 (11) Å	θ = 1–27.5°
*c* = 8.3440 (14) Å	µ = 0.10 mm^−^^1^
β = 113.02 (3)°	*T* = 150 K
*V* = 1140.4 (7) Å^3^	Needle, colorless
*Z* = 4	0.90 × 0.17 × 0.12 mm

### Data collection

**Table:**

Bruker–Nonius KappaCCD area-detector diffractometer	2522 independent reflections
Radiation source: fine-focus sealed tube	1547 reflections with *I* > 2σ(*I*)
graphite	*R*_int_ = 0.139
Detector resolution: 9.091 pixels mm^-1^	θ_max_ = 27.5°, θ_min_ = 3.0°
φ and ω scans	*h* = −5→6
Absorption correction: gaussian (Coppens, 1970)	*k* = −39→35
*T*_min_ = 0.961, *T*_max_ = 0.993	*l* = −10→9
7864 measured reflections	

### Refinement

**Table:**

Refinement on *F*^2^	Primary atom site location: structure-invariant direct methods
Least-squares matrix: full	Secondary atom site location: difference Fourier map
*R*[*F*^2^ > 2σ(*F*^2^)] = 0.111	Hydrogen site location: inferred from neighbouring sites
*wR*(*F*^2^) = 0.274	H-atom parameters constrained
*S* = 1.13	*w* = 1/[σ^2^(*F*_o_^2^) + (0.0574*P*)^2^ + 2.6221*P*] where *P* = (*F*_o_^2^ + 2*F*_c_^2^)/3
2522 reflections	(Δ/σ)_max_ < 0.001
145 parameters	Δρ_max_ = 0.33 e Å^−^^3^
0 restraints	Δρ_min_ = −0.41 e Å^−^^3^

### Special details

**Table:**

Geometry. All e.s.d.'s (except the e.s.d. in the dihedral angle between two l.s. planes) are estimated using the full covariance matrix. The cell e.s.d.'s are taken into account individually in the estimation of e.s.d.'s in distances, angles and torsion angles; correlations between e.s.d.'s in cell parameters are only used when they are defined by crystal symmetry. An approximate (isotropic) treatment of cell e.s.d.'s is used for estimating e.s.d.'s involving l.s. planes.
Refinement. Refinement of *F*^2^ against ALL reflections. The weighted *R*-factor *wR* and goodness of fit *S* are based on *F*^2^, conventional *R*-factors *R* are based on *F*, with *F* set to zero for negative *F*^2^. The threshold expression of *F*^2^ > σ(*F*^2^) is used only for calculating *R*-factors(gt) *etc*. and is not relevant to the choice of reflections for refinement. *R*-factors based on *F*^2^ are statistically about twice as large as those based on *F*, and *R*- factors based on ALL data will be even larger.

### Fractional atomic coordinates and isotropic or equivalent isotropic displacement parameters (Å^2^)

**Table:**

	*x*	*y*	*z*	*U*_iso_*/*U*_eq_	
O1	0.5287 (7)	0.28398 (13)	0.5987 (6)	0.0682 (12)	
O2	−0.4491 (7)	0.08832 (12)	0.3236 (4)	0.0538 (9)	
O3	0.3626 (7)	0.04751 (12)	0.8379 (5)	0.0544 (10)	
N1	0.0720 (8)	0.30545 (13)	0.5611 (6)	0.0468 (10)	
H1	−0.0993	0.2975	0.5571	0.056*	
N2	0.1165 (9)	0.34981 (14)	0.5371 (6)	0.0548 (11)	
H2A	0.2856	0.3587	0.5404	0.066*	
H2B	−0.0256	0.3683	0.5186	0.066*	
C1	0.2785 (10)	0.27498 (16)	0.5905 (7)	0.0469 (12)	
C2	0.1853 (14)	0.22916 (18)	0.6146 (10)	0.074 (2)	
H2	0.0374	0.2236	0.6559	0.089*	
C3	0.3475 (11)	0.19327 (16)	0.5674 (7)	0.0516 (13)	
H3	0.5095	0.1979	0.5373	0.062*	
C4	0.2192 (10)	0.14822 (15)	0.5752 (6)	0.0437 (11)	
C5	−0.0544 (10)	0.13674 (16)	0.4480 (6)	0.0451 (11)	
H5	−0.1530	0.1561	0.3573	0.054*	
C6	−0.1797 (9)	0.09645 (15)	0.4560 (6)	0.0408 (10)	
C7	−0.0380 (9)	0.06710 (16)	0.5864 (6)	0.0417 (10)	
H7	−0.1233	0.0400	0.5912	0.050*	
C8	0.2389 (10)	0.07918 (15)	0.7127 (6)	0.0404 (10)	
C9	0.3672 (9)	0.11891 (15)	0.7090 (6)	0.0391 (10)	
H9	0.5512	0.1261	0.7945	0.047*	
C10	−0.5868 (11)	0.0472 (2)	0.3233 (7)	0.0605 (15)	
H10A	−0.4515	0.0237	0.3308	0.073*	
H10B	−0.7611	0.0443	0.2179	0.073*	
H10C	−0.6420	0.0461	0.4217	0.073*	
C11	0.6609 (10)	0.05504 (18)	0.9613 (7)	0.0547 (13)	
H11A	0.7879	0.0602	0.8999	0.066*	
H11B	0.7292	0.0296	1.0344	0.066*	
H11C	0.6652	0.0802	1.0318	0.066*	

### Atomic displacement parameters (Å^2^)

**Table:**

	*U*^11^	*U*^22^	*U*^33^	*U*^12^	*U*^13^	*U*^23^
O1	0.0387 (18)	0.061 (2)	0.117 (4)	−0.0048 (17)	0.043 (2)	0.001 (2)
O2	0.0457 (18)	0.065 (2)	0.0394 (19)	−0.0032 (17)	0.0038 (15)	−0.0008 (16)
O3	0.0441 (18)	0.056 (2)	0.049 (2)	−0.0058 (16)	0.0027 (15)	0.0103 (16)
N1	0.0296 (18)	0.050 (2)	0.062 (3)	−0.0042 (17)	0.0198 (18)	−0.005 (2)
N2	0.043 (2)	0.049 (2)	0.072 (3)	0.0035 (19)	0.022 (2)	0.009 (2)
C1	0.040 (2)	0.050 (3)	0.056 (3)	−0.005 (2)	0.024 (2)	−0.003 (2)
C2	0.076 (4)	0.048 (3)	0.134 (6)	−0.010 (3)	0.080 (4)	−0.008 (3)
C3	0.048 (3)	0.046 (3)	0.069 (4)	0.000 (2)	0.032 (3)	0.006 (2)
C4	0.043 (2)	0.047 (3)	0.049 (3)	−0.003 (2)	0.026 (2)	−0.004 (2)
C5	0.048 (3)	0.051 (3)	0.035 (2)	0.008 (2)	0.015 (2)	0.008 (2)
C6	0.038 (2)	0.049 (3)	0.037 (2)	0.002 (2)	0.0157 (19)	−0.006 (2)
C7	0.040 (2)	0.043 (2)	0.043 (3)	−0.005 (2)	0.017 (2)	−0.004 (2)
C8	0.042 (2)	0.045 (3)	0.035 (2)	0.003 (2)	0.0155 (19)	0.0017 (19)
C9	0.033 (2)	0.045 (3)	0.038 (2)	−0.0019 (19)	0.0119 (18)	−0.0043 (19)
C10	0.043 (3)	0.078 (4)	0.049 (3)	−0.008 (3)	0.006 (2)	−0.009 (3)
C11	0.046 (3)	0.064 (3)	0.045 (3)	−0.003 (2)	0.007 (2)	0.006 (2)

### Geometric parameters (Å, °)

**Table:**

O1—C1	1.230 (5)	C4—C5	1.389 (7)
O2—C6	1.371 (5)	C5—H5	0.9301
O2—C10	1.416 (6)	C6—C5	1.381 (7)
O3—C8	1.374 (5)	C6—C7	1.367 (7)
O3—C11	1.438 (6)	C7—H7	0.9298
N1—N2	1.391 (6)	C8—C7	1.402 (6)
N1—C1	1.320 (6)	C8—C9	1.365 (6)
N1—H1	0.8600	C9—C4	1.389 (7)
N2—H2A	0.8601	C9—H9	0.9300
N2—H2B	0.8600	C10—H10A	0.9598
C1—C2	1.502 (7)	C10—H10B	0.9600
C2—H2	0.9300	C10—H10C	0.9600
C3—C2	1.489 (7)	C11—H11A	0.9601
C3—C4	1.517 (7)	C11—H11B	0.9600
C3—H3	0.9300	C11—H11C	0.9600
			
N2—N1—H1	118.2	C7—C6—C5	121.3 (4)
C1—N1—N2	123.6 (4)	O2—C6—C5	115.2 (4)
C1—N1—H1	118.2	C6—C7—C8	117.9 (4)
N1—N2—H2A	120.1	C6—C7—H7	120.9
N1—N2—H2B	119.9	C8—C7—H7	121.1
H2A—N2—H2B	120.0	C9—C8—O3	124.4 (4)
C6—O2—C10	117.8 (4)	C9—C8—C7	122.1 (4)
C8—O3—C11	117.1 (4)	O3—C8—C7	113.4 (4)
O1—C1—N1	121.8 (5)	C8—C9—C4	119.0 (4)
O1—C1—C2	123.1 (5)	C8—C9—H9	120.4
N1—C1—C2	115.1 (4)	C4—C9—H9	120.6
C1—C2—H2	122.6	O2—C10—H10A	109.9
C3—C2—C1	114.9 (4)	O2—C10—H10B	109.6
C3—C2—H2	122.5	O2—C10—H10C	108.9
C2—C3—C4	112.1 (4)	H10A—C10—H10B	109.5
C2—C3—H3	124.0	H10A—C10—H10C	109.5
C4—C3—H3	123.9	H10B—C10—H10C	109.5
C5—C4—C3	119.0 (4)	O3—C11—H11A	109.3
C5—C4—C9	119.8 (4)	O3—C11—H11B	109.3
C9—C4—C3	121.2 (4)	O3—C11—H11C	109.8
C4—C5—H5	120.1	H11A—C11—H11B	109.5
C6—C5—C4	119.9 (4)	H11A—C11—H11C	109.5
C6—C5—H5	120.0	H11B—C11—H11C	109.5
C7—C6—O2	123.6 (4)		

### Hydrogen-bond geometry (Å, °)

**Table:**

*D*—H···*A*	*D*—H	H···*A*	*D*···*A*	*D*—H···*A*
N1—H1···O1^i^	0.86	2.02	2.870 (3)	168.

Symmetry codes: (i) *x*−1, *y*, *z*.
